# Asthma-Associated COPD Etiotype: Clinical Features and Inflammatory Patterns in Biological Samples

**DOI:** 10.3390/jpm15120615

**Published:** 2025-12-10

**Authors:** Camyla Fernandez de Farias, José Baddini-Martinez, Ana Luisa Godoy Fernandes, Maria Marta Amorim, Michel Dracoulakis, Maria Amélia Santos, Lilian Ballini Caetano, Fernando Sergio Leitão Filho

**Affiliations:** Division of Respiratory Medicine, Department of Medicine, Universidade Federal de São Paulo, São Paulo 04024-002, Brazil

**Keywords:** asthma, pulmonary disease, chronic obstructive, sputum, nasal lavage, interleukin-8

## Abstract

**Background**: The Global Initiative for Chronic Obstructive Lung Disease (GOLD) recognizes asthma as a potential causal pathway for chronic obstructive pulmonary disease, referred to as the COPD-A etiotype. However, the clinical and inflammatory characteristics of this phenotype remain poorly defined. **Objectives**: This study aimed to characterize clinical traits and cytokine profiles in stable asthmatics exhibiting persistent airflow limitation compatible with COPD-A. **Methods**: In this cross-sectional study, 94 stable asthmatic patients (71.3% female; age = 54.0 ± 15.6 years) without relevant smoking or environmental exposures were evaluated. COPD-A was defined by a post-bronchodilator FEV_1_/FVC ratio < 0.70. Asthma control (ACQ, ACT), quality of life (AQLQ), and lung function were assessed. Levels of IL-5, IL-8, IL-13, IL-17A, IL-17F, IL-25, IL-33, and TNF were quantified in nasal lavage, induced sputum, and blood samples. **Results**: Among the participants, 42 (44.7%) fulfilled COPD-A criteria. Compared with non-COPD-A subjects, those with COPD-A were older (60.5 vs. 48.7 years; *p* < 0.001) and had longer disease duration (39.8 vs. 30.1 years; *p* < 0.001), lower post-bronchodilator FEV_1_% predicted (68.1 vs. 87.1%; *p* < 0.001), and poorer asthma control (ACQ = 1.00 vs. 0.64; *p* = 0.003). Cytokine levels were comparable between groups except for higher IL-8 concentrations in induced sputum of COPD-A subjects (7.66 vs. 2.51 pg/mL; *p* = 0.024). Sputum IL-8 ≥ 3.096 pg/mL independently predicted COPD-A (aOR = 12.82; *p* = 0.023). **Conclusions**: Over 40% of non-smoking asthmatics exhibited persistent airflow limitation consistent with COPD-A. Elevated sputum IL-8 levels may be a potential biomarker of this etiotype.

## 1. Introduction

Asthma is defined by the Global Initiative for Asthma (GINA) as a heterogeneous disease, typically characterized by chronic airway inflammation, airway hyperresponsiveness, variable respiratory symptoms, and reversible expiratory airflow limitation [1]. In contrast, the Global Initiative for Chronic Obstructive Lung Disease (GOLD) guidelines describe COPD as a heterogeneous condition characterized by chronic respiratory symptoms and progressive, persistent airway limitation, primarily caused by abnormalities of the airways and alveoli (emphysema) [2]. Distinguishing between these two respiratory diseases can be challenging, and they might coexist in the same patient under the umbrella concept of asthma–COPD overlap (ACO), particularly among current and former smokers [3]. Recent advances in our understanding of COPD pathogenesis have emphasized that smoking, despite being its leading cause, does not account for all COPD cases [4]. Consistent with this notion, a novel etiotype-based taxonomy aimed at classifying COPD according to its underlying causal mechanisms has been proposed [2,4]: COPD-G (genetic), COPD-D (abnormal lung development), COPD-C (cigarettes), COPD-P (pollution), COPD-I (infection), COPD-A (asthma), COPD-U (unknown cause), and COPD-M (mixed causes). This taxonomy intends to better capture real-world heterogeneity and support more individualized clinical management [2,4,5]. Within this framework, COPD-A refers to COPD arising as a consequence of long-standing asthma, differentiating it conceptually and mechanistically from the old ACO construct, which grouped together patients with mixed or coexisting features without a defined etiological pathway.

The relationship between asthma and COPD remains complex and incompletely understood [1,2,3,4]. Evidence suggests that airway hyperresponsiveness, even in people without established asthma, increases the risk of developing COPD [6]. Self-reported asthma has been linked to accelerated decline in FEV_1_ in population-based cohorts [7]. In addition, in a prospective study, individuals with asthma exhibited a 12-fold higher risk of developing COPD over 20 years, even after adjustment for smoking history, supporting asthma as a contributor to COPD pathogenesis [8].

A central mechanism connecting asthma to COPD-A would be airway remodeling (AR), a process driven by persistent, uncontrolled airway inflammation [9]. AR results from interactions among structural airway cells, immune cells, and a network of inflammatory mediators. Among these mediators, alarmins (IL-25, IL-33, and thymic stromal lymphopoietin) have emerged as upstream regulators linking epithelial injury to downstream type 2 and non-type 2 inflammatory cascades. Other cytokines implicated in remodeling include IL-4, IL-5, IL-13, IL-17 family cytokines, eotaxin, periostin, transforming growth factor-β, and extracellular matrix components [10,11,12,13]. These mediators promote goblet cell hyperplasia, increased smooth muscle mass, basement membrane thickening, angiogenesis and subepithelial fibrosis, ultimately leading to progressive and sometimes irreversible airflow limitation. Notably, features of AR have been identified even in preschool children with severe recurrent wheezing, suggesting that airway remodeling may begin early in life and progress over subsequent decades [14].

Since persistent airflow limitation is a hallmark of both advanced asthma with AR and COPD, it is reasonable to hypothesize that asthmatics exhibiting chronic structural airway changes may represent the clinical substrate of the COPD-A etiotype [2,4]. However, despite this plausible link, the clinical characteristics and inflammatory signatures of COPD-A are still poorly defined. Previous studies have not provided a comprehensive assessment of cytokine profiles across multiple airway compartments in well-characterized patients with long-standing asthma and minimal or no smoking exposure.

To address this knowledge gap, the present study undertook a detailed investigation of clinical traits and cytokine profiles in nasal lavage (NL), induced sputum (IS), and plasma obtained from adult asthma patients with minimal or no smoking history. Participants were stratified according to the presence or absence of persistent post-bronchodilator (BD) airflow limitation, which was employed as a functional surrogate for the COPD-A etiotype. Building on previous work from our group demonstrating that NL can partially mirror lower airway inflammatory processes, analyses across all three biological matrices were performed to enable a more comprehensive and integrated characterization of the inflammatory signatures associated with COPD-A.

## 2. Materials and Methods

### 2.1. Subjects

Patients with a confirmed diagnosis of asthma were consecutively recruited from the adult asthma outpatient clinic of the Respiratory Division at the Federal University of São Paulo (Unifesp), Brazil. All participants were required to be clinically stable, defined as the absence of respiratory tract infections, acute asthma exacerbations, or need for changes in controller or rescue medications within the preceding three months. Exclusion criteria were as follows: (i) known severe systemic comorbidities or pulmonary diseases other than asthma; (ii) pre-bronchodilator FEV_1_ < 40% of predicted, to ensure the safety of induced sputum procedures; (iii) smoking history ≥ 10 pack-years; (iv) any tobacco use within the preceding 12 months; and (v) chronic biomass exposure, defined as ≥5 years of near-daily exposure to indoor cooking fires or wood-burning stoves, or prolonged occupational biomass smoke exposure. The last three criteria were implemented to ensure that any airflow limitation observed would not be plausibly attributable to biomass or smoke-related airway injury.

All patients were using long-acting beta-agonists combined with inhaled corticosteroids (ICS) as maintenance therapy. No patient was using oral corticosteroids or any biologic agent.

The study protocol was approved by the Unifesp Research Ethics Board (CAAE: 47579915.5.0000.5505, approved on 17 November 2015), and all volunteers provided written informed consent.

### 2.2. Proceedings

At the screening visit, eligible subjects were instructed to discontinue nasal corticosteroids and topical decongestants for 48 h and ICS for 24 h prior to the second visit. Although the effects of ICS may persist beyond 24–48 h, extending the washout period further would raise ethical concerns in patients with moderate to severe asthma. The interval adopted reflects a pragmatic compromise, previously implemented at our center, that minimizes potential interference with cytokine measurements while avoiding undue risk of exacerbation [15,16].

On the day of evaluation, quality-of-life impairment was quantified using the validated Asthma Quality of Life Questionnaire (AQLQ) [17]. Disease control was assessed using two standardized instruments: the Asthma Control Test (ACT) and the Asthma Control Questionnaire (ACQ) [18,19].

Spirometry, pre- and post-BD, was performed using a Koko USB spirometer (Ferraris, Louisville, CO, USA), following the Brazilian Thoracic Association guidelines [20]. Bronchodilator responsiveness was evaluated 15 min after administering 400 µg of salbutamol (albuterol, GSK, Rio de Janeiro, Brazil). A post-BD increase ≥7% of predicted in FVC or FEV_1_ was considered significant [20]. Persistent airflow limitation was defined as a post-BD FEV_1_/FVC ratio < 0.70, and patients meeting this criterion were classified as belonging to the COPD-A etiotype [2,4]

NL was collected from all participants before sputum induction and processed as previously described [15,16]. Briefly, 5 mL of sterile 0.9% saline (22–28 °C) was instilled into each nostril with the head tilted 30° backward. Participants held their breath and performed a Valsalva maneuver for 10 s, then leaned forward to expel the fluid into a sterile container. At least 7 mL was obtained and stored at 4 °C until processing within one hour. Participants then underwent sputum induction by inhaling increasing concentrations of hypertonic saline (3%, 4%, and 5%) for 7 min at each concentration through a mouthpiece without a valve or nose clip [21,22]. For safety, spirometry was performed immediately after each inhalation. The criterion adopted for interrupting sputum induction was an FEV_1_ fall ≥ 20% from the post-BD baseline value. All participants completed each 7 min inhalation period without adverse events. IS samples were collected in sterile containers and processed within 1 h. 

Nasal and IS samples were shaken vigorously and centrifuged for 15 min at 2500× *g* and 4 °C. The fluid phases were separated and kept refrigerated. The cell pellets were resuspended in 0.5 mL of phosphate-buffered saline, and the total number of cells was determined using a Neubauer hemocytometer (Labor, São Paulo, Brazil). Cell suspensions of 200 µL were spun at 36× *g* for 6 min in a Cytospin 4 Shandon Centrifuge (Thermo Fisher Scientific, New York, NY, USA), and the resulting cell smears were stained with Panotics Wright’s Stain Kit (Laborclin, Paraná, Brazil). A total of 200 cells were analyzed for differential cell count.

Lastly, blood samples were drawn from participants and centrifuged to separate the plasma. All samples (NL, IS, and plasma) were subsequently stored in −80 °C freezers until the time of analysis.

### 2.3. Biochemical Analyses

NL and IS samples were completely dried using a SpeedVac concentrator (Savant SC200, Refrigerated Vapor Trap RT400; Thermo Fisher Scientific, Waltham, MA, USA) for 4 h and subsequently reconstituted in 150 μL of distilled water just before analyzing.

Levels of IL-5, IL-8, IL-13, IL-17A, IL-17F, IL-25, IL-33, and tumor necrosis factor (TNF) were measured simultaneously in all clinical samples using the bead-based multiplex immunoassay technique with MILLIPLEX^®^ kits (HTH17MAG-14K-07, HCYTOMAG-60K-01, HSTCMAG-28SK-01; Merck KGaA, Darmstadt, Germany) according to the manufacturer’s recommendations. Plate readings were taken on a Bio-Plex^®^ 200 System (Bio-Rad Laboratories, Hercules, CA, USA), which employs Luminex xMAP technology.

### 2.4. Statistical Analyses

Continuous numerical variables were expressed as mean ± standard deviation (SD) or as median with minimal and maximal values when appropriate, whereas categorical variables were presented as counts and percentages. Normality of distributions was assessed using the Shapiro–Wilk test. Comparisons of numerical variables between the COPD-A and non-COPD-A groups were performed using Student’s *t*-test or Mann–Whitney U test for normally and non-normally distributed data, respectively. Proportions related to categorical variables from both groups were compared using Pearson’s Chi-square test. Logistic regression analyses were performed to identify potential risk factors associated with COPD-A etiotype, and results were expressed as adjusted odds ratios (aOR) with 95% confidence intervals (CI). Statistical significance was set at *p* < 0.05 (two-tailed). Missing samples were excluded from the analyses, a pragmatic and commonly accepted approach in exploratory studies, particularly when the sample size is modest. No correction for multiple comparisons was applied because most analyses were hypothesis-driven and focused on predefined cytokines of biological relevance, and adjusting *p*-values in this context could substantially increase the risk of type II error in a modest sample size. All analyses were performed using the SPSS software (version 25.0; Chicago, IL, USA).

## 3. Results

A total of 94 asthmatic patients were prospectively evaluated in this study. The mean age was 54 ± 15.6 years, and 67 (71.3%) were females. Eighty-five (90.4%) were never-smokers, eight had a cumulative tobacco exposure of 5 pack-years or less, and one reported a smoking history of 7 pack-years. No volunteer reported significant previous or current biomass smoke exposure (Table 1).

According to the spirometry findings, 42 participants (44.7%) were classified as COPD-A and 52 (55.3%) were included in the non-COPD-A group (Table 1). Both groups showed similar proportions of females (non-COPD-A: 75.0%; COPD-A: 66.7%; *p* = 0.49) and ever-smokers (non-COPD-A: 7.7%; COPD-A: 11.9%; *p* = 0.51). In contrast, subjects in the COPD-A group were significantly older (mean age 63.7 vs. 51.8 years; *p* < 0.001), had a longer duration of asthma (median 39.8 vs. 30.1 years; *p* < 0.001), and were using higher daily doses of inhaled budesonide compared with their counterparts (median of 1200 μg vs. 800 μg; *p* = 0.014). Despite no significant differences in asthma control or asthma-related quality of life (assessed by ACT and AQLQ scores, respectively), the median ACQ score was higher in the COPD-A group, indicating poorer asthma control (1.00 vs. 0.64; *p* = 0.003). Consistent with this, patients with COPD-A showed a trend toward greater asthma severity, with 50% receiving GINA step 5 treatment, compared with 32.7% in the non-COPD-A group (*p* = 0.097). The prevalence of allergic rhinitis was higher among non-COPD-A participants (59.6% vs. 40.4%; *p* = 0.021).

The COPD-A group demonstrated significantly lower pre-BD FVC and FEV_1_ values, expressed in both absolute terms and as percentage of predicted, as well as lower FEV_1_/FVC ratio, compared with the non-COPD-A group (*p* < 0.05 for all comparisons) (Table 2). Post-BD results showed that the COPD-A group had a significantly greater increase in FVC (median absolute variation: +160 mL; median change as percentage of predicted: +5.0%), in contrast to +50 mL (*p* = 0.003) and +1.0% (*p* = 0.002), respectively, in the non-COPD-A group. As a result, post-BD FVC did not differ significantly between the groups either in absolute values (COPD-A group: 2.91 ± 0.89 L vs. non-COPD-A group: 3.18 ± 0.79 L, *p* = 0.12) or percentage of predicted (COPD-A group: 88.7 ± 15.2% vs. non-COPD-A group: 93.3 ± 12.0%, *p* = 0.10). Conversely, post-bronchodilator FEV_1_ remained significantly lower in the COPD-A group compared with the non-COPD-A group: 1.78 ± 0.59 L (68.1 ± 12.8% of predicted) vs. 2.44 ± 0.64 L (87.1 ± 11.2% of predicted), respectively (*p* < 0.001 for absolute and predicted values). Notably, according to current Brazilian Thoracic Society recommendations [20], 54.8% of individuals in the COPD-A group demonstrated bronchodilator responsiveness (based on either FVC or FEV_1_ increase) during spirometry, compared with 25.0% in the non-COPD-A group (*p* = 0.005).

A few technical issues prevented the measurement of inflammatory markers in some samples across the different clinical specimens (Table 3, Table 4 and Table 5). Overall, the cytological and cytokine profiles, including IL-5, IL-13, IL-17A, IL-17F, IL-25, IL-33, and TNF levels, did not differ significantly between the groups in either NL, IS, or blood samples. Despite the relatively small dataset, IL-8 levels in IS were significantly higher in the COPD-A group compared with the non-COPD-A group (median 7.66 vs. 2.51 pg/mL; *p* = 0.024), a finding not replicated in nasal lavage or plasma.

Based on these findings, we next performed logistic regression analyses adjusted for multiple covariates (age, sex, body mass index, asthma duration, ACQ score, post-bronchodilator FEV_1_ [% of predicted], and IS IL-8 levels categorized as <3.096 pg/mL vs. ≥3.096 pg/mL, using the overall median as the cutoff) to identify independent risk factors for the COPD-A etiotype (Table 6). Among all variables included in the model, only post-bronchodilator FEV_1_ (aOR = 0.84; 95% CI: 0.74–0.95; *p* = 0.006) and IS IL-8 levels ≥ 3.096 pg/mL (aOR = 12.82; 95% CI: 1.41–116.47; *p* = 0.023) remained strongly and independently associated with the COPD-A etiotype.

## 4. Discussion

The continuous advancement in the field of COPD underscores that even its definition remains an evolving construct. COPD is now recognized as a heterogeneous condition that may arise from causal pathways beyond tobacco exposure. Within this context, the newly proposed COPD-A taxonomy offers a potentially useful framework for identifying patients whose persistent airflow limitation derives predominantly from asthma-related mechanisms, rather than the classic smoking-induced injury. In contrast to ACO, which denotes the coexistence of clinical features from both conditions, COPD-A represents a distinct etiological trajectory. This distinction might contribute to a more refined characterization of risk factors, inflammatory profiles, and therapeutic responses, thereby supporting a more accurate understanding of the biological diversity encompassed within the COPD spectrum.

The present study aimed to investigate the clinical and inflammatory characteristics of a well-defined group of asthmatic patients with minimal or no smoking history and without significant environmental exposures who align with the proposed concept of COPD-A. Most strikingly, we found that nearly half of the study participants (42/94, 44.7%), who were being followed in an asthma clinic at a tertiary hospital, also met spirometric criteria (post-bronchodilator FEV_1_/FVC ratio < 0.7) consistent with a diagnosis of COPD-A [2,4]. It is important to emphasize that all subjects were free of any respiratory condition other than asthma and had been clinically stable for at least three months prior to enrollment. This criterion is clinically relevant, as patients undergoing asthma exacerbations could have been misdiagnosed as having the COPD-A etiotype due to transitory severe airflow limitation on spirometry. The prevalence of COPD-A observed in this cohort was somewhat higher than the 35.2% reported among 579 COPD patients recruited from the Korean Obstructive Lung Disease (KOLD) cohort [23]. A factor likely contributing to this discrepancy is that our cases were identified among moderate and severe asthma patients under longitudinal follow-up, whereas the Korean study used a cross-sectional design focused primarily on individuals with established COPD. In this study, and consistent with previous observations, individuals classified as COPD-A were older and reported a longer history of asthma symptoms [23,24,25]. These findings underscore the potential contribution of such factors to the development of COPD-A. A longer history of asthma implies prolonged periods of chronic airway inflammation, which increases the risk of AR and may ultimately lead to persistent airflow limitation. In the KOLD cohort referenced above, after adjustments for age and body mass index, the authors found that asthma was an independent risk factor for COPD (aOR: 5.92 (95% CI: 3.97–8.85); *p* < 0.001) [23]. Aging, in turn, is associated with substantial changes in both the cellular and extracellular components of the airways, as well as in their immunological and inflammatory responses. Such changes contribute to elevated risks of lung cancer, respiratory infections, interstitial lung diseases, and COPD in older people, although the mechanisms underlying these aging-related processes remain the focus of ongoing investigation [26,27]. In this context, the observed association between COPD-A and older age may reflect broader aging-related biological processes rather than being solely attributable to asthma. Other factors that may also interact with asthma and potentially increase the risk of developing the COPD-A etiotype include occupational exposures, maternal smoking, childhood respiratory tract infections, and a history of airway hyperresponsiveness originating in early life [25].

The COPD-A group naturally exhibited significantly worse lung function than the non-COPD-A group, as the groups were defined based on a post-bronchodilator FEV_1_/FVC ratio < 0.7. Beyond lower pre-bronchodilator FVC, FEV_1_, and FEV_1_/FVC values, COPD-A participants demonstrated a greater BD-induced increase in FVC and more frequently met the Brazilian bronchodilator responsiveness criteria [28]. Notably, post-BD FVC values did not differ between the groups, despite the significantly lower post-BD FEV_1_ mean observed in COPD-A participants. These patterns suggest a greater degree of baseline air trapping in the COPD-A group, which was partially reversed after bronchodilation. In addition, the generally good level of asthma control across both groups may have limited the magnitude of bronchodilator-induced changes among the participants.

Higher asthma severity was also observed among subjects in the COPD-A group. Consistent with this, we noted a trend toward a greater proportion of subjects receiving GINA step 5 treatment in the COPD-A group (50.0% vs. 32.7%). To achieve asthma control, COPD-A subjects were also prescribed significantly higher daily inhaled doses of budesonide compared with non-COPD-A subjects. The COPD-A group also demonstrated a higher median ACQ score (1.00, indicating borderline control) than the non-COPD-A group (0.64, indicating satisfactory control), whereas ACT scores were comparable between groups. Taken together, these findings suggest that the ACQ may be a more sensitive instrument than the ACT for evaluating asthma control in patients with the COPD-A etiotype.

The panel of cytokines evaluated was selected to capture the major inflammatory pathways believed to contribute to persistent airflow limitation in COPD-A. IL-5 and IL-13 represent type-2-mediated eosinophilic inflammation, which has been implicated in airway remodeling in long-standing asthma. IL-8 is a potent neutrophil chemoattractant strongly associated with fixed airflow obstruction and structural airway changes. IL-17A and IL-17F reflect type-17-driven neutrophilic responses linked to corticosteroid resistance and chronic inflammation. IL-25 and IL-33 act as epithelial alarmins that amplify type 2 inflammation and may contribute to the progression from reversible to persistent obstruction. TNF, a pleiotropic pro-inflammatory cytokine, has also been associated with airway remodeling and loss of lung function across obstructive airway diseases. Together, these mediators encompass complementary biological pathways relevant to COPD-A, providing a strong rationale for their inclusion in this investigation.

The results showed that COPD-A and non-COPD-A subjects exhibited broadly similar cytokine profiles across NL, IS, and plasma samples, except for IS IL-8. These results should not be interpreted as evidence that the evaluated cytokines play no meaningful role in asthma-related airway inflammation. On the contrary, it is highly plausible that key mediators such as IL-5 and IL-13 remain central to type-2-driven airways inflammatory responses in asthmatic individuals, irrespective of the presence of the COPD-A etiotype. The lack of between-group differences may only indicate that both phenotypes share fundamental inflammatory pathways intrinsic to asthma, rather than suggesting a lack of biological activity of these cytokines. Notably, the inclusion of a non-asthmatic control group would have allowed clearer discrimination between normal patterns, asthma-specific inflammatory responses and those potentially modulated by fixed airflow limitation.

The notable exception was IS IL-8, which was significantly higher in the COPD-A group (7.66 vs. 2.51 pg/mL). This finding was reinforced by logistic regression analyses, in which elevated sputum IL-8 (≥3.096 pg/mL) was associated with a 12-fold greater likelihood of meeting COPD-A criteria. Given that asthma predominantly affects the airways and that sputum is a more direct reflection of this microenvironment than nasal or blood specimens, the association between COPD-A and higher IL-8 levels is biologically plausible. IL-8 is a key mediator in several inflammatory pathways; it is produced by macrophages, epithelial cells, and endothelial cells and acts as a potent chemoattractant and activator of neutrophils [29]. Its central role in COPD-related neutrophilic inflammation is well characterized, whereas asthma is classically linked to eosinophilic and lymphocytic responses [2,29,30]. The elevation of IL-8 observed in COPD-A subjects suggests that this cytokine in the sputum may have potential as a predictive or diagnostic biomarker for this etiotype. Nevertheless, this hypothesis requires further replication and validation in larger and externally diverse cohorts.

This study has several limitations, including its cross-sectional design, convenience sample size, and the recruitment of all participants from a single center. There were also technical barriers that hindered the determination of certain measurements in multiple samples, consequently diminishing the statistical power of the study.

Cytological and biochemical measurements were missing for different reasons. In several patients, insufficient NL or IS volumes precluded proper processing, and in some cases, the resuspended sputum was excessively viscous, occasionally obstructing the analytical apparatus. In addition, several cytokine values were unavailable despite the use of a standardized bead-based multiplex assay. This pattern reflects known analyte-specific variability in airway samples rather than methodological failure [31,32]. Low-abundance cytokines (e.g., IL-5, IL-13, IL-25, IL-17A/F) frequently fell below the lower limit of detection, whereas IL-8, in many cases, most likely exceeded the upper quantification range at the dilution optimized for low-level markers. Matrix effects related to viscosity, proteolytic activity, and variable cellularity may also have influenced detection. Overall, the missing data reflect sample limitations and biological concentration extremes rather than systematic assay errors.

Additionally, as the washout phase for nasal and ICS was limited to 24 and 48 h before cytokine measurements, respectively, it is unclear whether a longer discontinuation period could have provided different results from those observed. Nonetheless, we should point out that the study population consisted primarily of moderate and severe asthmatics, and longer washout of ICS could have led to an increased risk of asthma exacerbations among the participants, thus raising potentially ethical concerns. Similarly, these washout periods have been used in a previous study from our center, and no issues were raised by the authors when performing similar experiments [16]. In addition, the present analyses should not be extrapolated to severe asthma patients, as those with severely low FEV_1_ (<40% of predicted) were excluded from participation due to safety concerns related to the administration of hypertonic saline for IS collection. Finally, the lack of correction for multiple comparisons may have increased the risk of type I error, although the analyses were based on predefined, biologically relevant cytokines.

The cross-sectional design of this study does not allow for the establishment of a causal relationship between asthma and the development of COPD. Nevertheless, this association is considered highly plausible based on current evidence in the literature, and the findings of the present study contribute modestly to a more precise characterization of features related to the COPD-A etiotype.

## 5. Conclusions

In conclusion, among clinically stable asthma patients without significant smoking history or environmental exposures, more than 40% exhibited persistent airflow limitation consistent with the COPD-asthma etiotype. These individuals were older, had longer asthma duration, and showed higher IL-8 levels in IS. Such findings indicate that a subset of asthmatics may develop fixed airflow limitation despite the absence of classical risk factors. The greater air trapping observed in the COPD-A group suggests that structural airway changes may develop over time, supporting the need for closer monitoring in patients at increased risk. The association between elevated IS IL-8 levels and the COPD-A phenotype supports a relevant role for this mediator, as well as for neutrophilic airway inflammation, in the development of this condition. This observation also raises the possibility that IL-8 may serve as a biomarker of inflammatory activity in this subgroup, although validation in larger, longitudinal studies is still required. Overall, recognition of the COPD-A etiotype may enhance patient stratification and foster more personalized approaches to disease management in COPD.

## Figures and Tables

**Table 1:** Clinical characteristics of the study population.

Variables	Non-COPD-A (n = 52)	COPD-A (n = 42)	*p*-Value
Sex (n, %)	F: 39 (75) M: 13 (25)	F: 28 (66.7) M: 14 (33.3)	0.49
Age (years)	48.7 ± 16.0	60.5 ± 12.4	<0.001
Ever-smokers (n, %)	4 (7.7)	5 (11.9)	0.51
BMI (kg/m^2^)	27.7 ± 5.1	26.5 ± 3.8	0.19
Asthma onset age (years)	15.5 (1–62)	19.5 (1–56)	0.23
Asthma lifetime history (years)	30.1 (5.0–68.4)	39.8 (17.4–71.3)	<0.001
Allergic rhinitis (n, %)	31 (59.6)	14 (34.1)	0.021
GINA treatment step (n, %)	Step 3/4: 35 (67.3) Step 5: 17 (32.7)	Step 3/4: 21 (50.0) Step 5: 21 (50.0)	0.097
Daily inhaled budesonide (μg)	800 (200–1600)	1200 (400–1200)	0.014
ACT	23 (15–25)	23 (9–25)	0.64
ACQ	0.64 (0–2.71)	1.00 (0.43–3.71)	0.003
AQLQ	6.00 (3.45–7.00)	6.23 (2.88–7.00)	0.87

BMI = body mass index; ACT = Asthma Control Test; ACQ = Asthma Control Questionnaire; AQLQ = Asthma Quality of Life Questionnaire; COPD-A: Asthma-associated COPD etiotype; Non-COPD-A: Non asthma-associated COPD etiotype; F: female; M: male; GINA: Global Iniciative for Asthma.

**Table 2:** Lung function parameters of the study population.

Variables	Non-COPD-A (n = 52)	COPD-A (n = 42)	*p*-Value
FVC (L)	3.13 ± 0.81	2.72 ± 0.88	0.022
FVC (% predicted)	91.5 ± 11.7	82.6 ± 15.6	0.002
FEV_1_ (L)	2.33 ± 0.64	1.62 ± 0.57	<0.001
FEV_1_ (% predicted)	83.2 ± 11.0	62.2 ± 12.7	<0.001
FEV_1_/FVC (%)	74.6 ± 5.4	60.0 ± 7.8	<0.001
Post-BD FVC (L)	3.18 ± 0.79	2.91 ± 0.89	0.12
Post-BD FVC (% predicted)	93.3 ± 12.0	88.7 ± 15.2	0.10
Post-BD FEV_1_ (L)	2.44 ± 0.64	1.78 ± 0.59	<0.001
Post-BD FEV_1_ (% predicted)	87.1 ± 11.2	68.1 ± 12.8	<0.001
Post-BD FEV_1_/FVC (%)	76.6 ± 4.8	61.2 ± 8.0	<0.001
Delta (Post-Pre) FVC (mL)	+50.0 (−340 to +360)	+160.0 (−250 to +1400)	0.003
Delta (Post-Pre) FVC (% predicted)	+1.0 (−9.0 to +11.0)	+5.0 (−6.0 to +33.0)	0.002
Delta (Post-Pre) FEV_1_ (mL)	+90.0 (−230 to +480)	+125 (−70 to +1000)	0.29
Delta (Post-Pre) FEV_1_ (% predicted)	+3.0 (−7.0 to +19.0)	+6.0 (−3.0 to +30.0)	0.21
Positive Response to BD (n,%)	13 (25.0%)	23 (54.8%)	0.005

FVC = forced vital capacity; FEV_1_ = forced expiratory value in the first second; COPD-A: Asthma-associated COPD etiotype; Non-COPD-A: Non asthma-associated COPD etiotype; BD: bronchodilator.

**Table 3:** Cell counts and cytokine levels in nasal lavage of the study population.

Variables	Non-COPD-A (n = 50)	COPD-A (n = 39)	*p*-Value
Neutrophils (%)	45 (0–98)	30 (0–100)	0.31
Eosinophils (%)	2.5 (0.0–37.0)	1 (0–23)	0.38
Lymphomononuclear cells (%)	1 (0–17)	0 (0–12)	0.45
IL-5 (pg/mL)	0.37 (0.03–6.50)	0.32 (0–6.25)	0.18
IL-8 (pg/mL)	3.92 (0–100.21)	2.63 (0.11–33.45)	0.09
IL-13 (pg/mL)	8.82 (0–203.25)	8.06 (0–94.19)	0.30
IL-17A (pg/mL)	0.67 (0–37.11)	0.50 (0–8.32)	0.48
IL-17F (pg/mL)	0.008 (0–0.11)	0.009 (0–0.042)	0.99
IL-25 (pg/mL)	0.002 (0–0.042)	0 (0–0.011)	0.31
IL-33 (pg/mL)	1.59 (0–50.63)	0.60 (0–14.40)	0.14
TNF (pg/mL)	0.74 (0–8.42)	0.43 (0–3.65)	0.22

IL = interleukin; TNF = tumor necrosis factor.

**Table 4:** Cell counts and cytokine levels in induced sputum of the study population.

Variables	Non-COPD-A (n = 47)	COPD-A (n = 36)	*p*-Value
Neutrophils (%)	76 (0–98)	77.5 (0–99)	0.26
Eosinophils (%)	2 (0–40)	1.5 (0–36)	0.97
Lymphomononuclear cells (%)	0 (0–3)	0 (0–3)	0.78
IL-5 pg/mL (pg/mL) *	1.48 (0.13–6.57)	2.11 (0.06–33.75)	0.16
IL-8 pg/mL (pg/mL) **	2.50 (0.29–25.40)	7.66 (0.16–826.50)	0.024
IL-13 (pg/mL) ^+^	19.89 (0–136.38)	20.96 (0–166.35)	0.97
IL-17A (pg/mL) ^++^	0.76 (0–5.33)	1.30 (0–24.77)	0.25
IL-17F (pg/mL) ^++^	0.010 (0–0.045)	0.012 (0–0.064)	0.18
IL-25 (pg/mL) ^¶^	0.002 (0–0.009)	0.003 (0–0.019)	0.28
IL-33 (pg/mL) ^¶¶^	2.60 (0.13–9.25)	2.27 (0–37.28)	0.71
TNF (pg/mL) *	1.14 (0.07–5.44)	1.37 (0.07–115.21)	0.25

* Non-COPD-A, n = 39; COPD-A, n = 30; ** Non-COPD-A, n = 27; COPD-A, n = 17; ^+^ Non-COPD-A, n = 39; COPD-A, n = 29; ^++^ Non-COPD-A, n = 37; COPD-A, n = 28; ^¶^ Non-COPD-A, n = 38; COPD-A, n = 29; ^¶¶^ Non-COPD-A, n = 37; COPD-A, n = 27; IL = interleukin; TNF = tumor necrosis factor.

**Table 5:** Cell counts and cytokine levels in the blood of the study population.

Variables	Non-COPD-A (n = 50)	COPD-A (n = 40)	*p*-Value
Leucocytes (cells/mm)	6935 (4270–12,200)	7380 (4360–17,800)	0.25
Neutrophils (cells/mm)	4150.5 (1290–9638)	4385 (2143–14,774)	0.35
Eosinophils (cells/mm)	184 (0–1261)	198.5 (0–836)	0.88
IL-5 (pg/mL) *	4.83 (0–90.14)	4.42 (0–34.19)	0.18
IL-8 (pg/mL) *	9.48 (0–78.73)	8.76 (1.41–23.66)	0.97
IL-13 (pg/mL) *	770.61 (30.55–1495.47)	639.96 (151.71–1548.95)	0.30
IL-17A (pg/mL) *	1.65 (0–30.89)	1.39 (0–27.02)	0.33
IL-17F (pg/mL) *	0.02 (0–0.17)	0.01 (0–0.09)	0.11
IL-25 (pg/mL) *	0.01 (0–0.42)	0.00 (0–0.26)	0.31
IL-33 (pg/mL) *	0.00 (0–86.91)	0.00 (0–61.32)	0.30
TNF (pg/mL) *	14.17 (4.05–64.48)	16.49 (7.03–48.33)	0.27

* Non-COPD-A, n = 44; COPD-A, n = 35; IL = interleukin; TNF = tumor necrosis factor.

**Table 6:** Risk factors associated with the COPD-A etiotype in the study population.

Covariates	Univariable Analysis		Multivariable Analysis	
	Odds Ratio (95% CI)	*p*-Value	Adjusted Odds Ratio (95% CI)	*p*-Value
Age	1.06 (1.02–1.09)	<0.001	1.08 (0.99–1.17)	0.074
Sex female	0.67 (0.27–1.64)	0.376	1.43 (0.17–11.98)	0.739
BMI	0.94 (0.86–1.03)	0.202	0.84 (0.65–1.09)	0.194
Asthma duration	1.05 (1.02–1.08)	0.001	1.04 (0.98–1.11)	0.185
ACQ score	2.19 (1.12–4.30)	0.022	2.50 (2.47–13.27)	0.281
Post-BD FEV_1_ (% predicted)	0.86 (0.81–0.91)	<0.001	0.84 (0.74–0.95)	0.006
IS IL-8 ≥ 3.096 pg/mL	3.81 (1.22–11.91)	0.022	12.82 (1.41–116.47)	0.023

CI: confidence interval; BMI: Body Mass Index; ACQ: Asthma Control Questionnaire; BD: bronchodilator; FEV_1_: forced expiratory value in the first second; IS: induced sputum; IL: interleukin.

## Data Availability

The original contributions presented in this study are included in the article. Further inquiries can be directed to the corresponding author.

## Abbreviations

The following abbreviations are used in this manuscript:

ACO	Asthma–COPD Overlap
ACQ	Asthma Control Questionnaire
ACT	Asthma Control Test
aOR	Adjusted Odds Ratio
AQLQ	Asthma Quality Of Life Questionnaire
AR	Airway Remodeling
BD	Bronchodilator
BMI	Body Mass Index
CI	Confidence Interval
COPD	Chronic Obstructive Pulmonary Disease
FEV_1_	Forced Expiratory Value In The First Second.
FVC	Forced Vital Capacity
GINA	Global Initiative For Asthma
GOLD	Chronic Obstructive Pulmonary Disease
IL	Interleukin
IS	Induced Sputum
KOLD	Korean Obstructive Lung Disease
NL	Nasal Lavage
SD	Standard Deviation
TNF	Tumor Necrosis Factor
